# Impact of a structured teaching on the ill effects of tobacco chewing among Indian adults

**DOI:** 10.6026/973206300200731

**Published:** 2024-07-31

**Authors:** Sivasubramanian N., Mahalakshmi B., Jadav Hetvi Dilipkumar, Padma P., Makwana Dhara Kamleshbhai, Jamunarani P., Jamiraben Aasifmasud Mansuri, Macwan Ellis Bharatbhai

**Affiliations:** 1 Nootan College of Nursing, Sankalchand Patel University, Visnagar, Gujarat - 384315, India; 2 KMCH College of Nursing, Coimbatore, Tamilnadu - 641048, India

**Keywords:** Tobacco chewing, structured teaching program, health education, knowledge assessment

## Abstract

Tobacco chewing remains a prevalent health issue globally, particularly in India, where it is deeply ingrained in cultural practices. This study evaluates the impact of a structured teaching program on knowledge regarding the ill effects of tobacco chewing among adults enrolled in arts and commerce colleges in Patan, India. A quasi-experimental design was employed, with 100 participants recruited from arts and commerce colleges. The structured teaching program included educational sessions covering the harmful effects of tobacco chewing and cessation strategies. Pre and post-test knowledge assessments were conducted using a self-structured questionnaire. The majority of participants were aged 15-17 years (75%), male (75%), and from urban areas (58.33%). Significant improvements in knowledge scores were observed following the intervention (pretest mean score = 11.5, posttest mean score = 44.38), with a significant difference between pre and post-test scores (t = 11.38, p < 0.001). Associations between pre-test knowledge scores and demographic variables such as gender, education, type of family, area of residence, father's education and occupation, monthly income, history of illness, and previous knowledge were identified (p < 0.05). Conclusion: The study underscores the significance of targeted health education programs in addressing tobacco-related health risks and promoting public health. By enhancing awareness and knowledge among adults, such interventions contribute to fostering behavior change and reducing the burden of tobacco-related diseases.

## Background:

Tobacco use, including chewing, remains a significant public health challenge globally, contributing to a wide range of adverse health outcomes and placing a substantial burden on healthcare systems. According to the World Health Organization [WHO], tobacco kills more than 8 million people worldwide each year, with over 7 million of these deaths attributed to direct tobacco use and approximately 1.2 million to non-smokers exposed to secondhand smoke. Despite widespread awareness of the harmful effects of tobacco, its use continues to be prevalent, particularly in certain regions and population groups. [1] In India, where tobacco consumption is deeply ingrained in cultural and social practices, chewing tobacco is a common form of use, especially among adults. The Global Adult Tobacco Survey [GATS] conducted in India revealed that approximately 28.6% of adults [aged 15 and above] use tobacco in some form, with smokeless tobacco products, including chewing tobacco, being the most prevalent. This high prevalence underscores the urgent need for targeted interventions to address tobacco use and its associated health risks effectively. [2] Among the myriad health consequences of tobacco use, oral health is particularly impacted by chewing tobacco. Studies have linked tobacco chewing to various oral conditions, including oral cancer, periodontal diseases, tooth decay, and mucosal lesions. [3] The carcinogenic components present in tobacco, such as nitrosamines and polycyclic aromatic hydrocarbons, increase the risk of oral cancer significantly. Additionally, the abrasive nature of tobacco products can damage oral tissues and contribute to gum recession and tooth loss. [4] Educational interventions play a crucial role in raising awareness about the harmful effects of tobacco use and promoting behavior change among individuals. Arts and commerce colleges provide a unique opportunity to implement such interventions, given their diverse student populations and emphasis on holistic education. By integrating health education programs into the curriculum, colleges can empower students with knowledge and skills to make informed decisions about their health and well-being. [5] Therefore, it is of interest to evaluate the effectiveness of a structured teaching program on knowledge regarding the ill effects of tobacco chewing among adults enrolled in selected arts and commerce colleges at Patna. The objectives include assessing baseline knowledge levels, measuring changes in knowledge following the intervention, and exploring associations between pre-test knowledge scores and demographic variables. Through rigorous evaluation and analysis, this study seeks to contribute valuable insights into the design and implementation of effective health education initiatives targeting tobacco use and its consequences.

## Methodology:

## Design:

A quasi-experimental design [6] was utilized to assess the effectiveness of a structured teaching program on knowledge regarding the ill effects of tobacco chewing among adults in selected arts and commerce colleges at Patna.

## Participants:

The study recruited 100 adults, including students and faculty members, from arts and commerce colleges in Patan.

## Intervention:

The structured teaching program consisted of educational sessions covering the harmful effects of tobacco chewing and cessation strategies, delivered through various methods such as lectures, discussions, and multimedia presentations.

## Data collection:

A pre-tested, self-structured questionnaire was used to collect demographic information and assess pre-test knowledge levels regarding tobacco chewing's ill effects. The same questionnaire was administered immediately after the intervention to assess post-test knowledge levels.

## Analysis:

Descriptive statistics summarized demographic characteristics and knowledge scores. Paired t-tests compared pre and post-test scores, and chi-square tests explored associations between knowledge scores and demographic variables [7].

## Results:

Table 1 summarizes the demographic characteristics of the sample. Majority of participants were aged 15-17 years [75%], male [75%], and from nuclear families [65%]. Most belonged to the Hindu religion [75%] and resided in urban areas [58.33%]. Additionally, a significant proportion had fathers employed as daily wage earners [55%] and mothers working as housewives [78%]. Comparison of pre and post scores among adults concerning the ill effects of tobacco chewing and its effects shows that the pretest means knowledge score was 11.5 with a standard deviation of 2.50; while the posttest means knowledge score was 44.38 with a standard deviation of 26.42. A paired t-test revealed a statistically significant difference between pre and post scores [t = 11.38, p < 0.001], indicating the effectiveness of the structured teaching program in enhancing knowledge regarding tobacco chewing and its effects. Associations between pre-test knowledge scores and demographic variables among adults shows there was Significant associations were found with gender (χ^2^ = 13.6), education (χ^2^ = 73.44), type of family (χ^2^ = 40.86), area of residence (χ^2^ = 47.55), father's education (χ^2^ = 59.58), father's occupation (χ^2^ = 84.81), monthly income (χ^2^ = 13.85), history of illness (χ^2^ = 13.6), and previous knowledge (χ^2^ = 47.55) (p < 0.05). No significant associations were observed for age, religion, mother's education, mother's occupation, and family history of tobacco chewing (p > 0.05)."

## Discussion:

The findings of this study reveal significant improvements in knowledge regarding the ill effects of tobacco chewing among adults following the structured teaching program. These results align with previous research highlighting the effectiveness of educational interventions in enhancing awareness of tobacco-related health risks. Comparing our study's results with those of several other studies, including Maya Sahu *et al.* (2017) and Baskaran *et al.* (2019), similar trends were observed in the post-test knowledge scores among participants [8,9]. Anshuman *et al.* (2019) also reported a notable increase in knowledge levels following a structured educational intervention focused on tobacco chewing. [10] These consistent findings underscore the importance of targeted health education programs in addressing gaps in knowledge and promoting behaviour change. Furthermore, the associations between pre-test knowledge scores and demographic variables identified in our study resonate with previous research. For instance, similar to our findings, studies conducted Chatterjee *et al.* (2014) found significant associations between knowledge levels and factors such as gender, education, and socioeconomic status [11]. This suggests that certain demographic characteristics may influence individuals' baseline knowledge levels and their receptiveness to educational interventions. Despite the promising results, our study has several limitations that warrant consideration. The use of convenience sampling may limit the generalizability of findings, and potential biases in self-reported data could affect the accuracy of results. Future research could employ larger, more diverse samples and utilize randomized controlled trial designs to further validate the effectiveness of educational interventions. In conclusion, the findings of this study highlight the efficacy of a structured teaching program in enhancing knowledge regarding tobacco chewing's ill effects among adults. By addressing gaps in knowledge and raising awareness of tobacco-related health risks, such interventions play a crucial role in promoting public health and fostering behavior change.

## Figures and Tables

**Figure 1:**
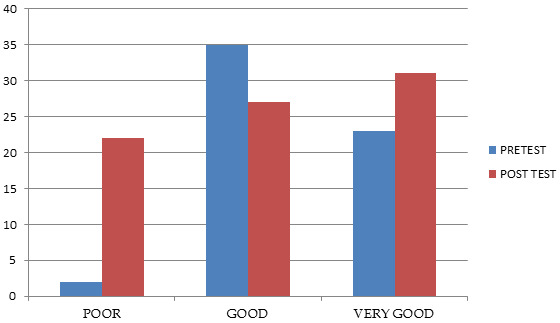
Showing distribution of sample as per their knowledge category

**Table 1:** Socio Demographic Data

**S. No.**	**Demographic Variables**	**Classification**	**Frequency**
1	Age in years	15-17	45
		18-20	4
		21-22	8
		>23	3
2	Gender	Male	45
		Female	15
3	Education	Undergraduate	35
		Postgraduate	25
4	Religion	Hindu	45
		Christian	4
		Muslim	8
		Others	3
5	Type of Family	Joint	18
		Nuclear	39
		Extended	3
6	Area of Residence	Rural	25
		Urban	35
7	Father's Educational Status	Illiterate	11
		Primary	9
		Secondary	16
		Graduate/Diploma	24
8	Mother's Educational Status	Illiterate	15
		Primary	17
		Secondary	14
		Graduate/Diploma	14
9	Father's Occupation	Unemployed	4
		Daily Wage Earner	33
		Self Employed	14
		Government	9
10	Mother's Occupation	Housewife	38
		Daily Wage Earner	10
		Self Employed	7
		Government	5
11	Monthly Income of the Family	5k - 10k	14
		10k - 15k	22
		15k - 20k	13
		above 20k	18
12	Family History of Tobacco Chewing	Yes	41
		No	19
13	History of any Illness in Past	Yes	40
		No	20
14	Previous Knowledge	Yes	39
		No	21
